# Can biochemical analyses risk stratify acutely admitted medical patients: an external validation of two existing systems

**DOI:** 10.1186/1757-7241-19-S2-P7

**Published:** 2012-04-16

**Authors:** Mikkel Brabrand, Torben Knudsen, Jesper Hallas

**Affiliations:** 1 Department of Medicine, Sydvestjysk Sygehus Esbjerg, Denmark; 2 Department of Clinical Pharmacology, University of Southern Denmark, Denmark

## Background

We sought to externally validate the ability of two existing risk stratification tools based primarily on biochemical analyses to predict in-hospital mortality. The systems were not restricted to specific presenting complaints or selected diagnoses.

## Methods

The study was completed as a prospective observational cohort study at a regional teaching hospital.

The discriminatory power (ability to discriminate between survivors and non-survivors) of the models was estimated using the area under the receiver-operating characteristics curve (AUROC). Values above 0.8 represent good discriminatory power. Calibration (accuracy of the prediction) was assessed using Hosmer-Lemeshow χ^2^ goodness of fit test with a p-value above 0.05 indicating acceptable calibration.

The blood tests used for calculation were drawn upon admission, and always within 2 hours of arrival at the department. As this was an observational study, only analyses ordered by the treating physician were included.

## Results

3,050 consecutively admitted medical patients were included. Eighty-four patients died while admitted (2.8 %).

When using the risk stratification system introduced by Prytherch et al. we were able to include 2,671 patients (87.6 %). We found a good discriminatory power with an AUROC of 0.852. Calculating the goodness of fit using the observed mortality data from the original article, we found a Hosmer-Lemeshow χ2 (eight degrees of freedom) of 570.5, p < 0.001.

Using the system introduced by Froom and Shimoni, we were only able to include 607 patients (19.9 %). However, we found an AUROC of 0.864 indicating a good discriminatory power. We were not able to calculate the goodness of fit using mortality data from the original article, as these were not provided. Thus using the predicted values in our cohort, we found a Hosmer-Lemeshow χ2 (eight degrees of freedom) of 71.7, p < 0.001.

## Conclusion

The two biochemical based risk stratification systems tested in this external prospective study were both able to predict in-hospital mortality with good discriminatory power but unacceptable calibration. However, we were only able to include 19.9 % of our cohort when calculating the score developed by Froom and Shimoni.

